# Genomic prediction for grain yield and micro-environmental sensitivity in winter wheat

**DOI:** 10.3389/fpls.2022.1075077

**Published:** 2023-02-01

**Authors:** Miguel A. Raffo, Beatriz C. D. Cuyabano, Renaud Rincent, Pernille Sarup, Laurence Moreau, Tristan Mary-Huard, Just Jensen

**Affiliations:** ^1^ Center for Quantitative Genetics and Genomics, Aarhus University, Aarhus, Denmark; ^2^ Université Paris Saclay, INRAE, AgroParisTech, GABI, Domaine de Vilvert, Jouy-en-Josas, France; ^3^ Génétique Quantitative et Evolution − Le Moulon, INRAE, CNRS, AgroParisTech, Université Paris-Saclay, Gif−sur−Yvette, France; ^4^ Nordic Seed A/S, Odder, Denmark; ^5^ Université Paris-Saclay, AgroParisTech, INRAE, UMR MIA-Paris Saclay, Palaiseau, France

**Keywords:** micro-environmental sensitivity, climatic resilience, genetic heterogeneity of residual variance, genomic selection, wheat

## Abstract

Individuals within a common environment experience variations due to unique and non-identifiable micro-environmental factors. Genetic sensitivity to micro-environmental variation (i.e. micro-environmental sensitivity) can be identified in residuals, and genotypes with lower micro-environmental sensitivity can show greater resilience towards environmental perturbations. Micro-environmental sensitivity has been studied in animals; however, research on this topic is limited in plants and lacking in wheat. In this article, we aimed to (i) quantify the influence of genetic variation on residual dispersion and the genetic correlation between genetic effects on (expressed) phenotypes and residual dispersion for wheat grain yield using a double hierarchical generalized linear model (DHGLM); and (ii) evaluate the predictive performance of the proposed DHGLM for prediction of additive genetic effects on (expressed) phenotypes and its residual dispersion. Analyses were based on 2,456 advanced breeding lines tested in replicated trials within and across different environments in Denmark and genotyped with a 15K SNP-Illumina-BeadChip. We found that micro-environmental sensitivity for grain yield is heritable, and there is potential for its reduction. The genetic correlation between additive effects on (expressed) phenotypes and dispersion was investigated, and we observed an intermediate correlation. From these results, we concluded that breeding for reduced micro-environmental sensitivity is possible and can be included within breeding objectives without compromising selection for increased yield. The predictive ability and variance inflation for predictions of the DHGLM and a linear mixed model allowing heteroscedasticity of residual variance in different environments (LMM-HET) were evaluated using leave-one-line-out cross-validation. The LMM-HET and DHGLM showed good and similar performance for predicting additive effects on (expressed) phenotypes. In addition, the accuracy of predicting genetic effects on residual dispersion was sufficient to allow genetic selection for resilience. Such findings suggests that DHGLM may be a good choice to increase grain yield and reduce its micro-environmental sensitivity.

## Introduction

Developing cultivars well adapted to the different environmental conditions where production is performed is one of the main goals of breeding programs. The performance of cultivars in different environments is affected by genotype-by-environment interactions (G×E). The G×E can result from environmental variation either due to differences in years and localities termed macro-environmental sensitivity, or from variability due to environmental disturbances within environments termed micro-environmental sensitivity. Selecting genotypes with lower macro and micro-environmental sensitivity is beneficial as they can present greater buffer capacity against external disturbances, thus contributing to a higher and more stable yield in a target population of environments. Given the global context of climate change and the expansion of agriculture to new areas, the development of climate-resilient varieties is also relevant to safeguarding the sustainability of wheat production (Tilman et al., 2011; Ray et al., 2013).

Micro-environmental sensitivity is defined as the genetic sensitivity to those environmental features that are specific to each individual (i.e. micro-environmental variation) and that cannot be identified or measured at each particular plot (Falconer and Mackay, 1996; Hill and Mulder, 2010; Walsh and Lynch, 2018). The micro-environmental sensitivity has been called in literature (confusingly) non-genetic variance, stochastic variation or general environmental variance, among other denominations (Falconer and Mackay, 1996; Dworkin, 2005; Morgante et al., 2015); nevertheless, several studies show that the micro-environmental sensitivity is partly under genetic control (Sorensen and Waagepetersen, 2003; Mulder et al., 2007; Ordas et al., 2008; Mulder et al., 2009; Vandenplas et al., 2013; Iung et al., 2017). The study of micro-environmental sensitivity has gained relevance in recent years for several animal species such as chickens (Wolc et al., 2009), pigs (Sorensen and Waagepetersen, 2003; Sell-Kubiak et al., 2015; Sell-Kubiak et al., 2022), sheep (SanCristobal-Gaudy et al., 1998; SanCristobal-Gaudy et al., 2001), cattle (Mulder et al., 2013a; Vandenplas et al., 2013), and aquaculture species (Sae-Lim et al., 2016). However, to the best of our knowledge, studies on micro-environmental sensitivity have barely been conducted in plants (e.g. Ordas et al., 2008 for maize), and have not been developed on commercial wheat varieties.

Before describing methods to estimate genetic variation in residual dispersion, it is useful to distinguish between biological (also seen as quantitative, Hill and Mulder, 2010) and statistical models used to infer genetic parameters. The ‘biological’ denotation refers to the theoretical genetic model specifying the genetic effects underlying the phenotype, while the ‘statistical’ sense refers to the model used to estimate genetic parameters related to these effects and is useful for predicting genetic values and estimating the response of selection (Falconer and Mackay, 1996). Several quantitative models explaining the influence of genetic effect on micro-environmental variance have been proposed in the literature (reviewed by Hill and Mulder, 2010); for example, the standard deviation model (Garcia et al., 2009), the additive model (Hill and Zhang, 2004; Mulder et al., 2007), and the exponential model (SanCristobal-Gaudy et al., 1998; SanCristobal-Gaudy et al., 2001; Sorensen and Waagepetersen, 2003). The exponential model, further described in the next section, has been proposed as a good alternative since the genetic values in micro-environmental variance are additive on a log scale, and it avoids the problem of having negative variances (Hill and Mulder, 2010; Walsh and Lynch, 2018).

Studying the genetic component of micro-environmental sensitivity is challenging as it implies modelling genetic effects at the level of residual dispersion (as a new trait). Different statistical methods to estimate variance components (VCs) and breeding values in micro-environmental variance have been proposed in animal breeding (SanCristobal-Gaudy et al., 1998; Sorensen and Waagepetersen, 2003; Rönnegård et al., 2010; Felleki et al., 2012; Iung et al., 2017). As a general distinction, those methods can be classified into a frequentist (likelihood-based) or a Bayesian framework. Sorensen and Waagepetersen (2003) applied a Bayesian methodology to estimate the genetic effects on the (expressed) phenotype and residual dispersion, as well as the genetic correlation between them. Among the main likelihood-based methods, Rönnegård et al. (2010) used a double hierarchical generalized linear model (DHGLM) developed by Lee and Nelder (2006). This methodology fits an algorithm that iterates between a linear mixed model at the level of (expressed) phenotypes and a generalized linear model (GLM) with gamma-distributed residuals at the level of residual dispersion. Felleki et al. (2012) extended the DHGLM model proposed by Rönnegård et al. (2010) to include the correlation between random genetic effects for (expressed) phenotypes and residual dispersion. The models proposed by Rönnegård et al. (2010) and Felleki et al. (2012) have been successfully applied since then and have been further extended to include genomic information and macro-environmental sensitivity, among other applications (Mulder et al., 2013a; Mulder et al., 2013b; Iung et al., 2017; Ehsaninia et al., 2019; Madsen et al., 2021; Sell-Kubiak et al., 2022). In addition, the DHGLM methodology has proven to be a computationally efficient approach to estimating micro-environmental sensitivity (Rönnegård et al., 2010; Felleki et al., 2012). Despite the availability of methodologies, common linear mixed models used for genomic prediction (GP) in plant breeding assume homogeneous residual variance within environments and do not account for micro-environmental sensitivity. Hence, more sophisticated methods are required to study micro-environmental sensitivity in plant breeding.

Several genetic parameters can be estimated to understand the implications of micro-environmental sensitivity in breeding; among the most relevant are the Mulder-Hill heritability of residual variance ( $h_{d}^{2}$ ), the genomic coefficient of variation of residual variance or evolvability (*GCV*_*E*_), and the correlation between additive genetic effects in (expressed) phenotypes and residual dispersion (*ρ*_*g*,*g*_*d*__). The $h_{d}^{2}$ is analogous to the narrow sense heritability in the classical sense (*h* ^2^ , Falconer and Mackay, 1996), and it is defined as the slope of the regression of the estimated additive values for dispersion on the squared phenotypes (*P* ^2^ , Mulder et al., 2007). The *GCV*_*E*_ indicates the proportion of micro-environmental variance that can be changed by selection, and the *ρ*_*g*,*g*_*d*__ allows to infer how the selection on additive genetic effects on (expressed) phenotypes will affect the additive genetic effects on dispersion. Estimates for these parameters can be obtained from the output of the DHGLM model and are useful references to develop more resilient cultivars exhibiting lower micro-environmental sensitivity. In addition, the $h_{d}^{2}$ and *GCV*_*E*_ can be useful for comparison across traits and species.

Plant species where inbred lines, single-crosses between inbred lines, or clones can be developed may present a high potential for studying micro-environmental sensitivity. That occurs because the availability of replications on the same individual contributes to a more accurate estimation of genetic variation and breeding values in micro-environmental variance (Vandenplas et al., 2013; Iung et al., 2017; Madsen et al., 2021). In this sense, studies on micro-environmental sensitivity in wheat can represent an opportunity, as highly homozygous inbred lines can be developed (e.g. six generations of selfing, F_6_), so that individuals originating from the same line can be considered genetically homogeneous. Another reason that makes wheat a valuable species for this study is that there has been broad literature reporting a considerable variation due to macro-environmental sensitivity (Bhatt and Derera, 1975; Cooper et al., 1995; Sial et al., 2000; Roozeboom et al., 2008; Lopez-Cruz et al., 2015; Crossa et al., 2017; Ly et al., 2017; Sukumaran et al., 2017; Ly et al., 2018; Crossa et al., 2021; Raffo et al., 2022a), and therefore, it encourages hypothesizing on a relevant genetic variation for micro-environmental sensitivity. In this study, we used a winter wheat breeding population phenotyped for grain yield, and we had two specific objectives:

1. To quantify the genetic variation on residual dispersion and, if it exists, the genetic correlation between genetic effects on (expressed) phenotype and residual dispersion for wheat grain yield using a double hierarchical generalized linear model (DHGLM).
2. To evaluate the predictive performance of the proposed double hierarchical generalized linear model for prediction of additive genetic effects on (expressed) phenotype and residual dispersion using cross-validation (CV) analysis.

To simplify reading, in the next sections we will refer to (expressed) phenotype as EGY (i.e. expressed grain yield) and to residual dispersion as DGY (i.e. residual dispersion of grain yield).

## Materials and methods

### Plant material

The data consisted of 2,456 sixth-generation (F_6_) winter wheat lines (*T. aestivum* L.) and 21,894 plot observations developed by Nordic Seed A/S breeding company. The breeding lines originated from nine breeding cycles (BC) tested in the years 2013 to 2021 (cycle 1: 2013–2014, cycle 2: 2014–2015, cycle 3: 2015–2016, cycle 4: 2016–2017, cycle 5: 2017, cycle 6: 2018, cycle 7: 2019, cycle 8: 2020, cycle 9: 2021) in three locations in Denmark (DK): Skive (north-west DK), Odder (central DK), and Holeby (south DK). In total, 26-year-location subsets were successfully assessed, and one failed (Odder, 2018) due to operational issues. The number of lines, plot observations, and average line replications per BC and for the whole population are presented together with descriptive statistics for grain yield in **Table 1**. Each BC came from approximately 60 parental line-crosses followed by selfing until F_6_, including family-based selection until third-generation (F_3_) and creating single seed descent (SSD) lines in the fourth-generation (F_4_). The experimental trials for each year-location combination comprised 15 blocks of 46 sowing plots of 8.25 m^2^, containing two replications of 21 F_6_ lines and two checks assigned randomly within each block. All lines were phenotyped for grain yield measured as kg per plot (8.25 m^2^). Agronomic practices were standardized within and across year-location subsets (e.g. application of disease treatments, fertilization).

**Table 1:** Descriptive statistics for the grain yield (kg grain/8.25m^2^) of F_6_ wheat breeding lines.

Breeding Cycle	Year	No. of Lines	No. of Plots	Average no. of line reps.	Average yield (SD)	Min. - Max Values	Coefficient of variation (%)
1	2013	78	1,060	13.59	8.89 (0.60)	6.48 – 10.45	6.70
2	2014	324	3,512	10.84	8.70 (0.76)	3.84 – 11.05	8.84
3	2015	232	2,574	11.09	8.64 (1.13)	4.75 – 11.47	13.11
4	2016	338	3,598	10.64	8.31 (1.37)	5.03 – 11.80	13.69
5	2017	160	1,012	6.32	9.13 (0.96)	6.21 – 11.40	10.58
6	2018	359	1,721	4.79	8.59 (0.46)	7.06 – 10.25	5.33
7	2019	315	2,589	8.22	9.38 (1.07)	6.04 – 12.36	11.38
8	2020	354	3,040	8.59	9.37 (0.68)	6.23 – 11.54	7.27
9	2021	305	2,788	9.14	9.02 (0.68)	6.45 – 11.32	7.55
Total	–	2,456	21,894	8.91	9.15 (0.82)	6.04 – 12.36	9.01

No.:, number; Reps., replications; SD, standard deviation; Min, Minimum; Max, Maximum.

### Genotyping

DNA were extracted based on a modified CTAB method (Rogers and Bendich, 1985). A 15K Illumina Infinium iSelect HD Custom Genotyping BeadChip technology (Wang et al., 2014) was used for genotyping. Quality control was performed by removing genotyped SNPs with a call rate lower than 0.90 and minor allele frequency (MAF) lower than 5%. Missing genotypes were imputed with the mean value after centering the genomic matrix (∼3% of missing values imputed). A total of 12,893 SNPs remained after the quality control.

### Corrected phenotypes

As a first step, a linear mixed model including all relevant effects for the population was utilized to analyze raw data. Such a model has been previously used in Raffo et al. (2022b) with a similar dataset from the same breeding program, and in brief, it was defined as: $\text{y}=\text{Xb}+\;{\text{Z}}_{1}\text{l}+{\text{Z}}_{2}\text{g}+\;{\text{Z}}_{3}\text{f}+\sum_{i=1}^{9}{\text{Z}}_{i+3}s+\text{e}$ , where ***Xb* , *Z*_1_ *l* , *Z*_2_ *g*** and ***Z*_3_ *f*** were defined as below in the LMM-HET (eq. 1), $\sum_{i=1}^{9}{\text{Z}}_{i+3}s$ is the spatial effect where *s* is the vector of spatial effect with $s\;\sim \;N\left(0,\text{I}\sigma_{s}^{2}\right)$ and $\sigma_{s}^{2}$ is the spatial effect variance, and *e* is a vector of random residuals with $\text{e }˜\;N\left(0,\text{I}\sigma_{\text{e}}^{2}\right)$ . The spatial effect contains the X and Y coordinate of the plot of the observation (i.e., target plot) and the eight surrounding plots (*n* = 9), and thus, it is the sum of the effects centered on those plots. A detailed description of the model and the spatial effect is given in Raffo et al. (2022b) under the heading “I+G_A_-model”. Second, the raw phenotypes were corrected by subtracting the estimates of the spatial effect (*s*) in order to correct for spatial variability within the experimental fields. The corrected phenotypes (***y*_*c*_**) were defined as: ***y*_*c*_** = ***y*−*Zs*** , where *y* represents the vector of raw phenotypes, and $\text{Z}=\sum_{i=1}^{9}{\text{Z}}_{i+3}$ . This allowed us to reduce the number of parameters to estimate in the DHGLM, facilitating convergence and decreasing computing time.

### Statistical analysis

#### Linear mixed model (LMM-HET)

A linear mixed model allowing for heteroscedasticity of residual variance for the different environments (hereinafter LMM-HET) was utilized in order to have a reference point for assessing the DHGLM. The LMM-HET was defined as:


(1) $${\text{y}}_{\text{c}}=\text{Xb}+{\text{Z}}_{1}\text{g}+{\text{Z}}_{2}\text{l}+{\text{Z}}_{3}\text{f}+{\text{e}}_{i}$$

where ***y*_*c*_** is a vector for the response variable EGY (i.e. corrected phenotypes) with 21,894 observations from the 2,456 lines, ***X*** and  ***Z*_*n*_** are the design matrices for fixed and random effects, respectively; *b* is a vector of fixed trial effect nested within year-location; *g* is a vector of additive genetic values with $\text{g}\;∼\;N\left(0,\text{G}\sigma_{\text{g}}^{2}\right)$ , $\sigma_{\text{g}}^{2}$ is the genomic additive variance, and ***G*** is the additive genomic relationship matrix (GRM) according to the first method of VanRaden (2008): $\text{G}=\frac{\text{Q}{\text{Q}}^{\prime }}{2\sum^{}p_{j}\left(1-p_{j}\right)}$ , where ***p*_*j*_** is the allele frequency of the *j* ^*th*^ *SNP*; ***Q*** is ***M*−*P***, with ***M*** as the SNP matrix coded -1, 0, 1, and ***P*** the matrix with the allele frequency of SNP *j* calculated as **1**(2(*p*_*i*_−0.5)) for column *j* ; *l* is a vector of line effect with $\text{l}\;∼\;N\left(0,\text{I}\sigma_{\text{l}}^{2}\right)$ , where ***I*** is an identity matrix and $\;\sigma_{\text{l}}^{2}$ is the variance due to uncorrelated line effects, which are common to all replicates of a line; *f* is a vector of line × environment interaction effect (L×E), with “E” defined for the different year-location subsets and $\text{f}\;∼\;N\left(0,\text{I}\sigma_{\text{f}}^{2}\right)$ , where $\sigma_{\text{f}}^{2}$ is the variance due to uncorrelated L×E effects; and *e*_*i*_ is a vector of random residuals with $\text{e}\;∼\;N\text{II}D\left(0,\text{I}\sigma_{{\text{e}}_{i}}^{2}\right)$ , where $\sigma_{{\text{e}}_{i}}^{2}$ is the residual heterogeneous variance fitted for the different year-location subsets (*i*=1, 2, …, 26).

The narrow (*h* ^2^) and broad-sense (*H* ^2^) heritabilities (Falconer and Mackay, 1996) at the plot level were estimated for LMM-HET for each year-location group (*i*) as: $h_{i}^{2}=d\left(\text{G}\right){\hat{\sigma }}_{\text{g}}^{2}/{\hat{\sigma }}_{{\text{P}}_{i}}^{2}$ and $H_{i}^{2}=\left({\hat{\sigma }}_{\text{l}}^{2}+d\left(\text{G}\right){\hat{\sigma }}_{\text{g}}^{2}\right)/{\hat{\sigma }}_{{\text{P}}_{i}}^{2}$ , where *d*(***G***) is the mean diagonal value of *G* with *d*(*G*)=1.84 , which can be interpreted as one plus the genomic inbreeding coefficient for the population (VanRaden, 2008); ${\hat{\sigma }}_{\text{g}}^{2}$ is the estimated genomic additive variance; $\;{\hat{\sigma }}_{\text{l}}^{2}$ is the estimated variance due to uncorrelated line effects; and ${\hat{\sigma }}_{{\text{P}}_{i}}^{2}$ is the estimated phenotypic plot variance for each year-location subset (subscript “ *i* “) calculated as: ${\hat{\sigma }}_{{\text{P}}_{i}}^{2}={\hat{\sigma }}_{\text{l}}^{2}+d\left(\text{G}\right){\hat{\sigma }}_{\text{g}}^{2}+{\hat{\sigma }}_{\text{f}}^{2}+{\hat{\sigma }}_{{\text{e}}_{i}}^{2}$ .

#### Double hierarchical generalized linear model

First, we start by briefly describing the exponential model (SanCristobal-Gaudy et al., 1998), which is the base quantitative model assumed in our study. The exponential model is an extension of the classical model (*P*=*μ*+*A*+*E*), and it was defined as: $P=\mu +\;A_{m}+L+F+\text{exp}[0.5\ln\left(\sigma_{E,\text{e}xp}^{2}\right)+0.5A_{v,\text{e}xp}$ ] *ϵ* , where *P* is the phenotype, *μ* is the intercept, *A*_*m*_ is the breeding value for the main effect, *L* is the polygenic genetic effect, *F* is the genetic × macro-environmnt interaction effect, exp specifies that the exponential function is used at the level of environmental variance, $\text{ln}\;(\sigma_{E,\text{e}xp}^{2}$ ) is the natural logarithm of the environmental variance, *A*_*v*,*exp*_ is the breeding value for the environmental variance, and *ϵ* is a scaled environmental deviation with variance one. As previously commented, in the exponential model the genetic values in environmental variance are additive on a log scale, and thus, it avoids the problem of having negative variances (Hill and Mulder, 2010; Walsh and Lynch, 2018).

The estimation of VCs and breeding values was performed running an iterative Average Information Restricted Maximum Likelihood (AI-REML) algorithm in DMU software (Madsen and Jensen, 2013) by the implementation of a DHGLM procedure (Lee and Nelder, 2006; Rönnegård et al., 2010; Felleki et al., 2012; Mulder et al., 2013a). The DHGLM was used to estimate genomic breeding values and genetic parameters for EGY and DGY. The DHGLM iterates using bivariate analysis (Equation 2), fitting a linear mixed model at the level of corrected phenotypes (***y*_*c*_**), and a Gamma GLM at the level of the dispersion variable *y*_*d*_ (*see* **Supplementary Material 1** *for an additional formal definition of the DHGLM*), where ${\text{y}}_{d_{n}}=\frac{{\hat{\text{e}}}_{n}^{2}}{1-\Lambda_{n}}$ , with ${\hat{\text{e}}}_{n}^{2}$ as the squared estimated residual for the *y*_*c*_*n*__ observation, and *Λ*_*n*_ is the ‘leverage’ defined as the diagonal element of the hat-matrix *H* (Hoaglin and Welsch, 1978) corresponding to *y*_*c*_*n*__ . The hat-matrix ***H*** is also known as the projection matrix since it provides ${\hat{\text{y}}}_{\text{c}}$ from ***y*_*c*_**as ${\hat{\text{y}}}_{\text{c}}=H^{*}{\text{y}}_{\text{c}}$ . The DHGLM is an algorithm fitting the exponential model and is given by Equation 2:


(2) $$\left[\begin{array}{c} {\text{y}}_{\text{c}}\\ {\text{y}}_{\text{d}} \end{array}\right]=\;\left[\begin{array}{c} \text{X}\\ 0 \end{array}\begin{array}{c} 0\\ \;{\text{X}}_{\text{d}} \end{array}\right]\;\left[\begin{array}{c} \text{b}\\ {\text{b}}_{\text{d}} \end{array}\right]+\left[\begin{array}{c} \;{\text{Z}}_{1}\\ 0 \end{array}\begin{array}{c} 0\\ \;{\text{Z}}_{2} \end{array}\right]\;\left[\begin{array}{c} \text{g}\\ {\text{g}}_{\text{d}} \end{array}\right]+\left[\begin{array}{c} \;{\text{Z}}_{3\;\;}\\ 0 \end{array}\right]\;\left[\text{l}\right]+\left[\begin{array}{c} \;{\text{Z}}_{4\;\;}\\ 0 \end{array}\right]\;\left[\text{f}\right]+\left[\begin{array}{c} \text{e}\\ {\text{e}}_{\text{d}} \end{array}\right]$$

where ***y*_*c*_** and ***y*_*d*_** are the vectors of response variables for EGY and DGY, respectively; ***X*** ,  ***b* , *Z*_*n*_ , *l*** , and ***f*** were defined as in LMM-HET. Note that ***l*** and ***f*** were included only for EGY since it was not possible for DGY due to problems of convergence in the REML algorithm. The convergence problems may be caused by a random effect with a variance close to 0, by between-trait correlations of -1 or 1, or by two different parameters in the model that are difficult to separate (i.e. high correlation between random effects for the same trait). ***X*_*d*_** is the design matrix for the fixed effects in DGY, ***b*_*d*_** is the vector of fixed trial effect nested within year-location in DGY; ***g*** and ***g*_*d*_** are the vectors of additive genetic values for EGY and DGY, respectively, following the distribution


$$\left[\begin{array}{c} \text{g}\\ {\text{g}}_{\text{d}} \end{array}\right]∼N\left(0,\;\left[\begin{array}{cc} \sigma_{g}^{2} & \sigma_{g,g_{d}}\\ \sigma_{g,g_{d}} & \sigma_{g_{d}}^{2} \end{array}\right]⊗\;\text{G}\right)$$

where $\sigma_{g}^{2}$ and $\sigma_{g_{d}}^{2}$ are the genomic additive variance for EGY and DGY, respectively, and *σ*_*g*,*g*_*d*__ is the covariance between genetics effect in EGY and DGY, ⊗ denotes the Kronecker product, and *G* the GRM as previously defined for the LMM-HET model; ***e*** and ***e*_*d*_** the vectors of random residual effects for EGY and DGY, respectively, following the distribution


$$\left[\begin{array}{c} \text{e}\\ {\text{e}}_{\text{d}} \end{array}\right]∼N\left(0,\;\left[\begin{array}{cc} {\text{W}}^{-1}\sigma_{e}^{2} & 0\\ 0 & {\text{W}}_{d}^{-1}\sigma_{\epsilon_{v}}^{2} \end{array}\right]\right)$$

where $\text{W}=diag{\left({\hat{\text{y}}}_{\text{d}}\right)}^{-1}$ and ${\text{W}}_{d}=diag\left(\frac{1-\Lambda }{2}\right)$ with *Λ* being the leverage (diagonal element of the hat-matrix ***H***), and $\sigma_{e}^{2}$ and $\sigma_{\epsilon_{v}}^{2}$ the scaling for residual variances constrained to have an average of one. Given that results on each trait of the bivariate model depend on each other, an iterative procedure was required to update ***W*** and ***W***_*d*_ until convergence of variance component estimates. Convergence was assumed when the relative difference in variance component estimates of two successive iterations was lower than 10^-5^. The bivariate model in Equation 2 was fitted using a reweighted least square (IRWLS) algorithm as follows:

1. Run a linear mixed model (LMM) with the same effects as in eq. 1 but assuming homoscedasticity of residual variance on ***y*_*c*_**.
2. Calculate ${\text{y}}_{d_{n}}={\hat{\text{e}}}_{n}^{2}/\left(1-\Lambda_{n}\right)$ , and ***W*_*d*_*n*__** =*diag*(1−*Λ*_*n*_)/2)
3. Run a weighted gamma generalized linear model (GLM) with a log-link function for response ***y*_*d*_** and weight ***W*_*d*_**
4. Calculate $W=dia\text{g}{\left({\hat{y}}_{d}\right)}^{-1}$

5. Run bivariate model in Equation 2
6. Update ***y*_*d*_, *W***, and ***W*_*d*_**
7. Iterates steps 3-6 until convergence

The model in Equation 2 and the iterative estimation procedure were developed by Felleki et al. (2012) and are based on a combination of the DHGLM proposed by Rönnegård et al. (2010) and the method proposed by Mulder et al. (2009). Note that convergence in step 5 is required to reach global convergence of the whole algorithm. The $h_{i}^{2}$ , $H_{i}^{2}$ , and $\sigma_{{\text{P}}_{i}}^{2}$ for EGY were estimated for the DHGLM using the equations described in the “*Linear mixed model (LMM-HET)*” section, but using an approximation for $\sigma_{{\text{e}}_{i}}^{2}$ computed as ${\hat{\sigma }}_{{\text{e}}_{i}}^{2}=\frac{\sum_{j=1}^{n}{\left(x_{ij}-\bar{x_{i}}\right)}^{2}}{n_{i}-1-npar_{i}}$ , where *x*_*ij*_ represents the residual for the *j* observation in the environment *i* , $\bar{x_{i}}$ is the mean value of residuals in the environment *i* ,  *n*_*i*_ is the number of observations in the environment *i* , and *npar*_*i*_ is the number of fixed effects estimated in the model for the environment *i* . Thus, the residual variance approximation is based on the variance of the final vector of residuals.

### Genetic parameters associated with micro-environmental sensitivity

Three genetic parameters were computed in order to interpret the micro-environmental sensitivity in our population: i) Mulder-Hill heritability of residual variance ( $h_{d}^{2}$ ), ii) genomic coefficient of variation of residual variance or evolvability (*GCV*_*E*_), and iii) genetic correlation between additive effects on mean and dispersion (*ρ*_*g*,*g*_*d*__). A description of the estimated parameters is provided next.

The $h_{d}^{2}$ can be defined (by analogy to *h* ^2^) as the regression of additive genetic values in dispersion on the squared phenotypes (*p* ^2^). The $h_{d}^{2}$ was estimated as defined by Mulder et al. (2007): $h_{d}^{2}=\frac{{\hat{\sigma }}_{{\text{g}}_{d,add}}^{2}}{2{\hat{\sigma }}_{\text{P}}^{4}+3{\hat{\sigma }}_{{\text{g}}_{d,add}}^{2}}$ , where ${\hat{\sigma }}_{{\text{g}}_{d,add}}^{2}$ is the rescaled genomic additive variance for DGY on the additive scale, and ${\hat{\sigma }}_{\text{P}}^{4}$ is the squared phenotypic variance. Note that for the estimation of $h_{d}^{2}$ , the additive genetic variance estimated for DGY required to be converted from the exponential scale $\left({\hat{\sigma }}_{{\text{g}}_{d}}^{2}\right)$ to the additive scale $\left({\hat{\sigma }}_{{\text{g}}_{d,add}}^{2}\right)$ . This conversion was performed using equations in Mulder et al. (2007): ${\hat{\sigma }}_{g_{d,add}}^{2}={\hat{\sigma }}_{e,exp}^{4}\exp({\hat{\sigma }}_{g_{d}}^{2})-{\hat{\sigma }}_{e,add}^{2}$ , where ${\hat{\sigma }}_{e,exp}^{2}=\frac{\left[\bar{\left(1/\text{W}\right)}{\hat{\sigma }}_{e}^{2}\right]}{\left[\exp(0.5{\hat{\sigma }}_{g_{d}}^{2})\right]}$ , ${\hat{\sigma }}_{g_{d}}^{2}$ is the additive genetic variance estimated for the residual variable on the exponential scale, ${\hat{\sigma }}_{g_{d}{,}_{add}}^{2}=\left[\bar{\left(1/\text{W}\right)}{\hat{\sigma }}_{e}^{2}\right]$ , with $\left[\bar{\left(1/\text{W}\right)}{\hat{\sigma }}_{e}^{2}\right]$ and ${\hat{\sigma }}_{e}^{2}$ the average of the reciprocal weights and the residual variance in the mean model, respectively (see Mulder et al., 2007 for complete derivations). The genetic coefficient of variation (*GCV_E_*) allows to infer how much the micro-environmental variance could be changed by selection (Mulder et al., 2016), and it was computed as $\text{G}CV_{E}=\sqrt{{\hat{\sigma }}_{{\text{g}}_{d}}^{2}}$ . The genetic correlation between additive effects on EGY and DGY (*ρ*_*g*,*g*_*d*__) was estimated as: $\rho_{\text{g},{\text{g}}_{d}}={\hat{\sigma }}_{\text{g},{\text{g}}_{d}}/\sqrt{{\hat{\sigma }}_{\text{g}}^{2}{\hat{\sigma }}_{{\text{g}}_{d}}^{2}}$ , with ${\hat{\sigma }}_{\text{g},{\text{g}}_{d}}$ , ${\hat{\sigma }}_{\text{g}}^{2}$ , and ${\hat{\sigma }}_{{\text{g}}_{d}}^{2}$ as previously defined for the DHGLM.

### Assessment of models and cross-validation

The predictive performances of the LMM-HET and the DHGLM were evaluated using the leave-one-line-out (LOO) cross-validation (CV) analysis. The LOO CV was performed by randomly masking the phenotypes of all replicates of one line and using the remaining lines to predict the additive genetic values. This process was repeated *t* -times (*t* = no. of lines = 2,456) until all lines were predicted.

Three estimates were computed to evaluate the predictive performance of the LMM-HET and the DHGLM models: i) Predictive ability (PA) calculated as the Pearson correlation (*ρ*) between the average value of lines after correcting for fixed effects and the vector of predicted additive genetic effects in EGY [ $\rho \left(\bar{{\text{y}}_{\text{c}}},\hat{\text{g}}\right)]$ and DGY [ $\rho \left(\bar{{\text{y}}_{\text{d}}},\hat{{\text{g}}_{\text{d}}}\right)]$ . The ${\bar{\text{y}}}_{\text{c}}$ and ${\bar{\text{y}}}_{d}$ were obtained by subtracting the fixed effects $\hat{\text{b}}$ and ${\hat{\text{b}}}_{\text{d}}$ estimated with the full dataset from each corresponding plot observation for EGY and DGY, respectively, and then averaging the resulting values by line. ii) The correlation between predicted additive genetic values obtained with “whole” phenotypic information for all lines and with “partial” phenotypic information (predictions for all lines when their own phenotypes were masked in the CV) was computed for EGY and DGY (***r***_*w*,*p*_ , Legarra and Reverter, 2018). iii) A statistic for variance inflation in predicted genetic values (***b***_*w*,*p*_) was estimated as the slope of the regression of predicted values obtained with whole phenotypic information on predicted values obtained with partial phenotypic information, ${\text{b}}_{w,p}=\;\frac{cov\left({\hat{g}}_{w},\;{\hat{g}}_{p}\right)}{var\left({\hat{\text{g}}}_{p}\right)}$ (Legarra and Reverter, 2018). An ordinary non-parametric bootstrap with replacement based on full sample size *m* = 2,456, and 10,000 replicates was used to obtain the standard error of the **PA, *r***_*w*,*p*_, and *b*_*w*,*p*_. A two-tailed paired t-test (critical *P-value* = 0.01) was used to compare PA,  *r*_*w*,*p*_ and *b*_*w*,*p*_ for the LMM-HET and DHGLM. The maximum potential PA for ***g*** predictions was computed for each year-location subset as $\sqrt{\text{k}h_{i}^{2}/\left(1+\left(\text{n}-1\right)h_{i}^{2}\right)}$ , where k was the average number of line repetitions and $h_{i}^{2}$ the heritability as previously defined, and then the average potential PA was calculated across the 26 year-location subsets. For ***g*_*d*_** the maximum potential PA was computed using the same formula but replacing $h_{i}^{2}$ with $h_{d}^{2}$ (note that it is an approximation as the Gamma distribution is not taken into account).

## Results

### Phenotypic data

**Figure 1:**
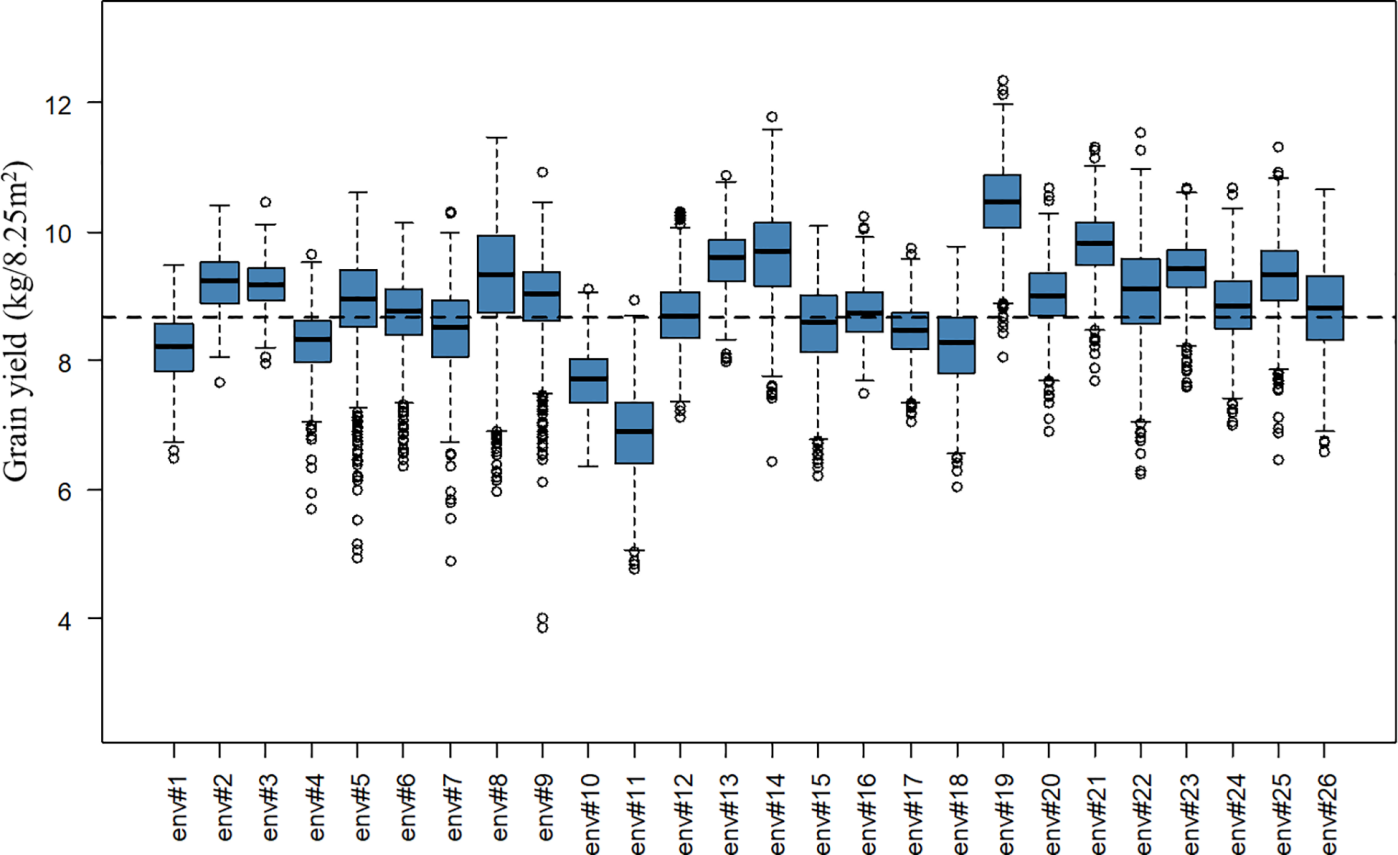
Boxplot of grain yield (kg/8.25m^2^) per year-location subset. The x-axis labels “env#1” to “env#26” indicates the 26 year-location environments where lines were tested. The black dashed line represents the overall average grain yield for the complete population.

A total of 2,456 F_6_ lines with an average number of replication of 8.91 were phenotyped for grain yield (kg grain/8.25m^2^), corresponding to 21,894 plot observations (see **Table 1** for descriptive statistics per BC and for the whole population). Note that for the interpretation of results in tons of grain/ha instead of kg grain/8.25m^2^, the presented values have to be multiplied by a conversion factor equal to 1.212. A considerable level of variation was observed for the different year-location subsets. The average grain yield varied from 6.90 to 10.45 kg grain/8.25m^2^ and the coefficients of variation varied from 4.01 to 10.38% for the different year-location subsets. A boxplot describing grain yield per year-location subset is presented in **Figure 1**.

### Variance components and heritability of additive genetic effect on mean

The proposed LMM-HET and DHGLM were utilized to estimate VCs on EGY and DGY, and the results are presented in **Table 2**.

**Table 2:** Variance components and heritability estimates for the linear mixed model allowing heteroscedasticity of residual variance for the different environments (LMM-HET) and the double hierarchical generalized linear model (DHGLM).

Models	LMM-HET									DHGLM									
										Mean model							Dispersion		Cor.
	${\hat{\sigma }}_{\text{g}}^{2}$	${\hat{\sigma }}_{\text{l}}^{2}$	${\hat{\sigma }}_{\text{f}}^{2}$	YL#	${\hat{\sigma }}_{\text{e}rror}^{2}$ ^†^	${\hat{\sigma }}_{\text{P}}^{2}$ ^†^	*h* ^2 †^	*H* ^2 †^	${\hat{\sigma }}_{\text{g}}^{2}$	${\hat{\sigma }}_{\text{l}}^{2}$	${\hat{\sigma }}_{\text{f}}^{2}$	YL#	${\hat{\sigma }}_{\text{e}rror}^{2}$ ^†^	${\hat{\sigma }}_{\text{P}}^{2}$ ^†^	*h* ^2 †^	*H* ^2 †^	${\hat{\sigma }}_{{\text{g}}_{d}}^{2}$ ^§^	$h_{d}^{2}$	$\rho_{{\hat{\sigma }}_{\text{g}}{\hat{\sigma }}_{{\text{g}}_{d}}}$
**Estimates**	0.054 (0.008)^*^	0.060 (0.004)	0.118 (0.003)	env#1	0.129	0.361	0.150	0.315	0.054 (0.007)	0.062 (0.004)	0.108 (0.003)	env#1	0.120	0.344	0.156	0.337	0.004 (0.004)	0.033	0.502
				env#2	0.080	0.311	0.173	0.365				env#2	0.105	0.329	0.163	0.352			
				env#3	0.038	0.269	0.201	0.423				env#3	0.114	0.338	0.158	0.343			
				env#4	0.079	0.310	0.174	0.366				env#4	0.110	0.334	0.160	0.347			
				env#5	0.114	0.346	0.156	0.329				env#5	0.105	0.329	0.163	0.352			
				env#6	0.068	0.299	0.181	0.380				env#6	0.110	0.335	0.160	0.346			
				env#7	0.106	0.337	0.160	0.337				env#7	0.072	0.297	0.181	0.391			
				env#8	0.348	0.579	0.093	0.196				env#8	0.083	0.307	0.174	0.377			
				env#9	0.054	0.285	0.189	0.399				env#9	0.104	0.329	0.163	0.353			
				env#10	0.036	0.267	0.202	0.425				env#10	0.124	0.348	0.154	0.333			
				env#11	0.099	0.330	0.164	0.345				env#11	0.107	0.331	0.162	0.350			
				env#12	0.072	0.304	0.178	0.374				env#12	0.102	0.327	0.164	0.355			
				env#13	0.064	0.296	0.183	0.385				env#13	0.132	0.356	0.150	0.325			
				env#14	0.077	0.308	0.175	0.369				env#14	0.104	0.328	0.163	0.353			
				env#15	0.067	0.298	0.181	0.381				env#15	0.107	0.331	0.162	0.350			
				env#16	0.063	0.294	0.183	0.386				env#16	0.050	0.274	0.195	0.422			
				env#17	0.078	0.309	0.175	0.368				env#17	0.048	0.272	0.197	0.426			
				env#18	0.064	0.296	0.183	0.385				env#18	0.054	0.278	0.193	0.417			
				env#19	0.088	0.319	0.169	0.357				env#19	0.057	0.281	0.190	0.412			
				env#20	0.081	0.312	0.173	0.365				env#20	0.053	0.277	0.193	0.418			
				env#21	0.046	0.278	0.195	0.410				env#21	0.055	0.279	0.192	0.416			
				env#22	0.335	0.566	0.095	0.201				env#22	0.053	0.277	0.193	0.418			
				env#23	0.048	0.280	0.193	0.407				env#23	0.050	0.274	0.195	0.423			
				env#24	0.090	0.321	0.168	0.354				env#24	0.066	0.290	0.185	0.399			
				env#25	0.087	0.318	0.170	0.357				env#25	0.070	0.294	0.182	0.394			
				env#26	0.082	0.313	0.173	0.363				env#26	0.063	0.287	0.187	0.404			
**Average**					0.096	0.327	0.171	0.359					0.085	0.310	0.175	0.377			
**Min.**					0.036	0.267	0.093	0.196					0.048	0.272	0.150	0.325			
**Max.**					0.348	0.579	0.202	0.425					0.132	0.356	0.197	0.426			

^*^ Values in round brackets are the standard deviation (SD) of estimates; **^†^** for estimates ${\hat{\sigma }}_{\text{e}rror}^{2}$ , ${\hat{\sigma }}_{\text{P}}^{2}$ , h^2^ , and H^2^ values are presented for the 26 year-location (YL#) subsets (“env#1” to “env#26”); Cor.: genetic correlation; **^§^** $\sigma_{{\text{g}}_{d}}^{2}$ result is in exponential scale. Min., Minumum; Max., Maximum.

A substantial amount of variance was observed in estimates for additive genetic variance ( ${\hat{\sigma }}_{g}^{2}$ ), line effects ( ${\hat{\sigma }}_{l}^{2}$ ) and line × environment interaction ( ${\hat{\sigma }}_{f}^{2}$ ) for EGY with both models. The ${\hat{\sigma }}_{g}^{2}$ had the same value for both models ( ${\hat{\sigma }}_{g}^{2}$ = 0.054). In general, small differences in estimates for ${\hat{\sigma }}_{l}^{2}$ and ${\hat{\sigma }}_{f}^{2}$ were observed between models, where the DHGLM had a slightly higher ${\hat{\sigma }}_{l}^{2}$ (0.062) compared to the LMM-HET (0.060), and lower ${\hat{\sigma }}_{f}^{2}$ (0.108) compared to the LMM-HET (0.118). The residual variance ( ${\hat{\sigma }}_{e_{i}}^{2}$ ) was obtained for the 26 different year-location subsets with both models (see **Table 2** for comparing specific year-location subsets). Generally, similar values for ${\hat{\sigma }}_{e_{i}}^{2}$ were observed between several of the 26-year-location subsets. The largest differences in ${\hat{\sigma }}_{e_{i}}^{2}$ were observed for environments #8 and #22 (env#8 and env#22 in **Table 2**), where the DHGLM had considerably lower ${\hat{\sigma }}_{e_{i}}^{2}$ (env#8: 0.083; env#22: 0.053) compared to the LMM-HET (env#8: 0.348 and env#22: 0.335).

The estimated phenotypic variance ( ${\hat{\sigma }}_{{\text{P}}_{i}}^{2}$ ), narrow-sense ( $h_{i}^{2}$ ) and broad-sense ( $H_{i}^{2}$ ) heritabilities were obtained for the 26-year-location subsets with both models (**Table 2**). The ${\hat{\sigma }}_{{\text{P}}_{i}}^{2}$ ranged from 0.267 to 0.579 with an average value of 0.327 for the LMM-HET, and from 0.272 to 0.356 with an average of 0.310 for the DHGLM. The $h_{i}^{2}$ varied across year-locations subsets from 0.093 to 0.202 for the LMM-HET and from 0.150 to 0.197 for the DHGLM. The average $h_{i}^{2}$ were 0.175 for the DHGLM and 0.171 for the LMM-HET. The $H_{i}^{2}$ ranged across year-locations from 0.196 to 0.425 for the LMM-HET and from 0.325 to 0.377 for the DHGLM, and the average $H_{i}^{2}$ was 0.377 for the DHGLM and 0.359 for the LMM-HET. The highest estimates for heritabilities were obtained for environments #10 for the LMM-HET and environment #17 for the DHGLM, and the lowest were observed for environments #8 for the LMM-HET and #13 for the DHGLM.

**Figure 2:**
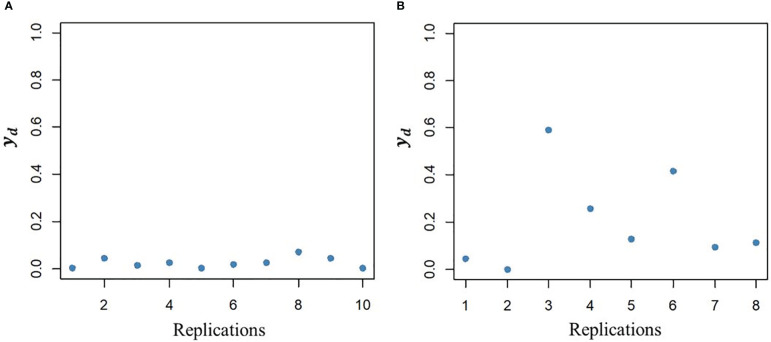
Illustration of variability between line replications in the dispersion variable (*y*_*d*_) after algorithm convergence for a line with low additive genetic value (**A**, left plot) and a line with high additive genetic value (**B**, right plot).

The DHGLM was used for the estimation of fixed effects ( ${\hat{\text{b}}}_{\text{d}}$ ) and additive genetic variance in DGY ( ${\hat{\sigma }}_{g_{d}}^{2}$ ). The ${\hat{\text{b}}}_{\text{d}}$ captured the variation due to trial effects nested within year-location at the level of DGY, and a substantial variation among ${\hat{\text{b}}}_{\text{d}}$ was observed (**Supplementary Material 2**, **Figure 1S**). The ${\hat{\sigma }}_{g_{d}}^{2}$ had a value of 0.004 (estimated in exponential scale), and it is reported in **Table 2**. The interpretation of ${\hat{\sigma }}_{g_{d}}^{2}$ , is linked to the estimation of genetic parameters associated with micro-environmental sensitivity, and thus   it was later used for the estimation of the Mulder-Hill heritability of residual variance ( $h_{d}^{2}$ ) and the genomic coefficient of variation of residual variance or evolvability (*GCV*_*E*_ , see next section: *“Genetic parameters estimated for micro-environmental sensitivity”*). The bivariate model in the DHGLM procedure allowed the estimation of the genetic correlation between additive genetic effects in EGY and DGY (*ρ*_*g*,*g*_*d*__ = 0.502). Further interpretation of *ρ*_*g*,*g*_*d*__ is presented in the following section. Differences in the predicted additive genetic values in DGY ( ${\hat{\text{g}}}_{d}$ ) were reflected in differences of within line variability, where extreme cases for lines with lower and higher ${\hat{\text{g}}}_{d}$ had low and high within line variation in DGY, respectively (**Figure 2**).

### Genetic parameters estimated for micro-environmental sensitivity

**Figure 3:**
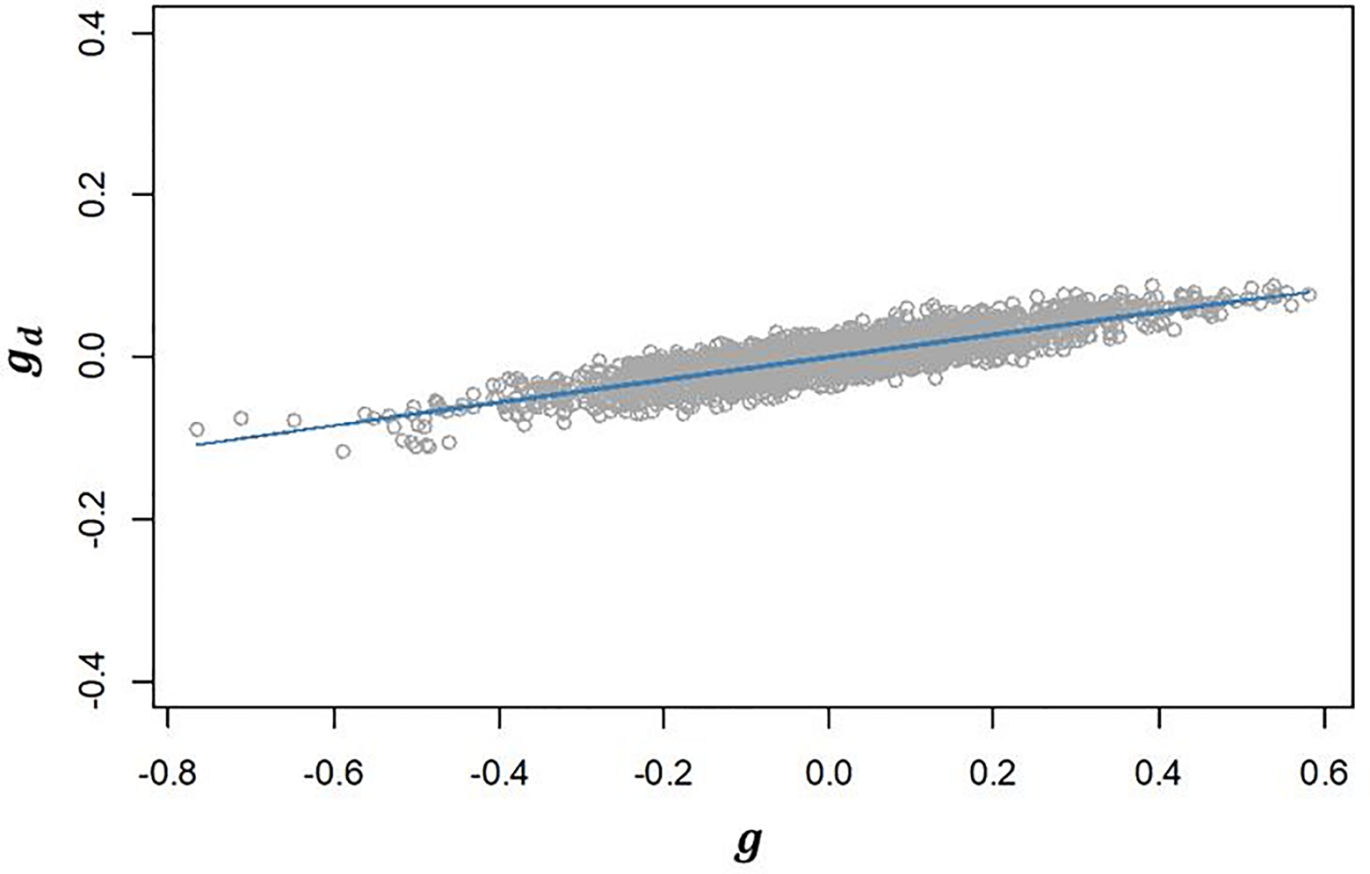
Regression of predicted additive genetic values for the dispersion variable (*g*_*d*_) after algorithm convergence on predicted additive genetic values for corrected phenotypes (*g*). The blue line represents the fitted curve of the linear regression.

The VCs presented in the previous section were utilized to estimate three genetic parameters associated with micro-environmental sensitivity ( $h_{d}^{2}$ , *GCV*_*E*_ , and *ρ*_*g*,*g*_*d*__):

- An estimate of Mulder-Hill heritability of residual variance ( $h_{d}^{2}$ , **Table 2**) of 0.033 was found for grain yield in our population. Such a value is situated at an intermediate level according to the range previously reported for other species, which varied from<0.01 to 0.10 (Wolc et al., 2009; Sonesson et al., 2013; Vandenplas et al., 2013; Sell-Kubiak et al., 2022).
- The genomic coefficient of variation of residual variance or evolvability (*GCV*_*E*_) found for our population was 0.061. The *GCV*_*E*_ is useful to infer the selection response in reducing micro-environmental variance when lines with lower additive values in DGY are selected (Rönnegård et al., 2013; Iung et al., 2020). Therefore, a *GCV*_*E*_ of 0.061 indicates that reducing the average additive values in DGY in one unit of *GCV*_*E*_ will reduce the micro-environmental variance by 6.1%.
- The genetic correlation between additive effects in EGY and DGY (*ρ*_*g*,*g*_*d*__) estimated for our population was 0.502 (**Table 2**). The positive correlation found suggests that selecting lines with higher additive values for $\hat{\text{g}}$ will have an effect on increasing the additive values for ${\hat{\text{g}}}_{d}$ and consequently will increase the micro-environmental sensitivity. This is confirmed by the strong linear relationship observed between $\hat{\text{g}}$ and ${\hat{\text{g}}}_{d}$ (**Figure 3** **).**

### Comparative predictive performance of LMM-HET and DHGLM

**Figure 4:**
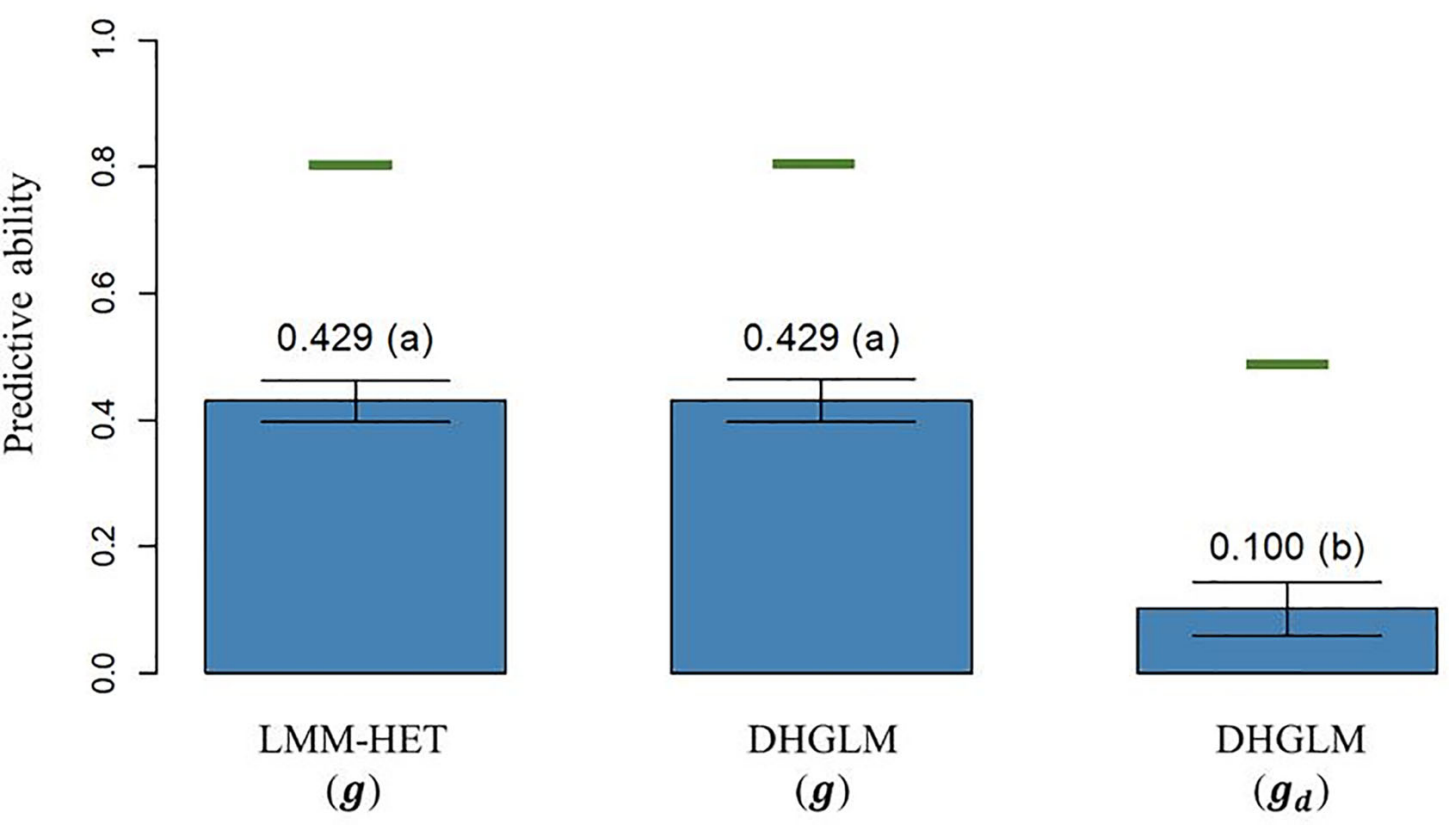
Predictive ability (PA) for the linear mixed model allowing heteroscedasticity of residual variance for the different environments (LMM-HET) and double hierarchical generalized linear model (DHGLM) in a leave-one-line-out (LOO) cross-validation. *g* : predicted additive genetic values for corrected phenotypes (*y*_*c*_). *g*_*d*_ : predicted additive genetic values for dispersion variable (*y*_*d*_). Black bars represent the 95% confidence interval (CI) computed for each estimate as the standard deviation of the estimate multiplied by 1.96. The letter above the bar denotes significant differences between models in a t-test (*P-value*< 0.01). The horizontal green lines represent the theoretical maximum PAs.

The predictive ability (PA), calculated as the Pearson correlation (*ρ*) between the average value of lines after correcting for fixed effects and the vector of predictions of the genetic additive effects on EGY [ $\rho \left(\bar{{\text{y}}_{\text{c}}},\hat{\text{g}}\right)]$ and DGY [ $\rho \left(\bar{{\text{y}}_{d}},\hat{{\text{g}}_{d}}\right)]$ obtained by LOO CV, is reported in **Figure 4**. The PA observed for predicted additive genetic values for EGY (predictions of ***g*** effect) in the DHGLM and the LMM-HET were 0.4292 and 0.4289, respectively, and no significant differences between models were observed in a bootstrap-based t-test (*P-value* > 0.01). The maximum potential PA for predictions of additive values for EGY (horizontal green lines in **Figure 4**) had similar values for both models, where the reported values were 0.807 for the DHGLM and 0.801 for the LMM-HET (average for the 26-year-location groups). The PA observed for predicted additive genetic values for   DGY (predictions of *g*_*d*_ effect) in the DHGLM was 0.1003, with a maximum potential PA of 0.483. The PA for DGY was significantly lower in the t-test (*P-value*< 0.01) than the PAs reported for EGY with the DHGLM and the LMM-HET.

**Figure 5:**
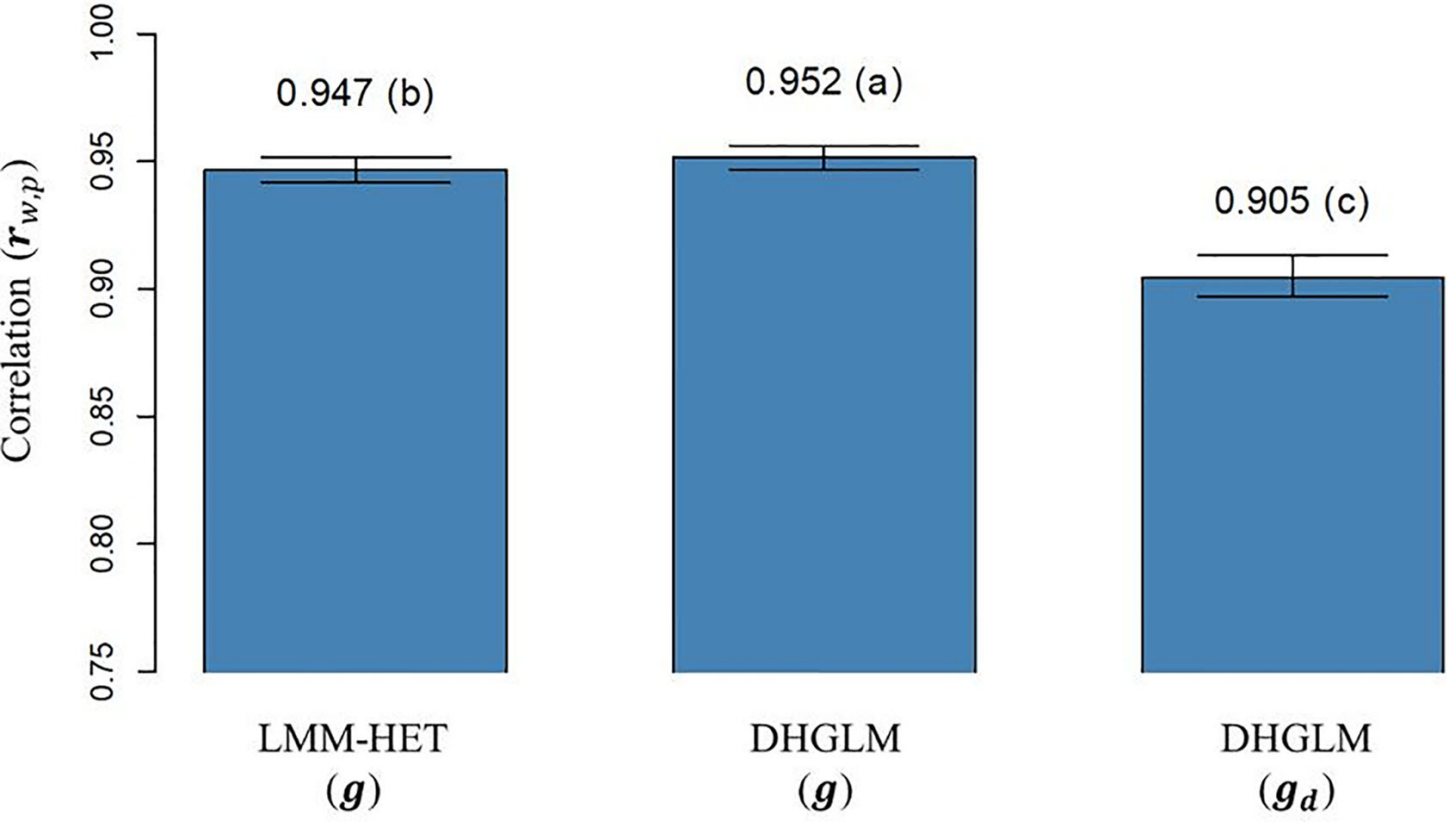
Correlation between predicted additive genetic values obtained with whole phenotypic information and additive values obtained with partial phenotypic information (*r*_*w*,*p*_) in a leave-one-line-out (LOO) cross-validation. LMM-HET: linear mixed model allowing heteroscedasticity of residual variance for the different environments. DHGLM: double hierarchical generalized linear model. *g* : predicted additive genetic values for corrected phenotypes (*y*_*c*_). *g*_*d*_ : predicted additive genetic values for dispersion variable (*y*_*d*_). Black bars represent the 95% confidence interval (CI) computed for each estimate as the standard deviation of the estimate multiplied by 1.96. The letter above the bar denotes significant differences between models in a t-test (*P-value*< 0.01).

The correlation (***r***_*w*,*p*_) between $\hat{\text{g}}$ (and ${\hat{\text{g}}}_{d}$ ) obtained with “whole” phenotypic information for all lines and “partial” phenotypic information from the LOO CV analysis is presented in **Figure 5**. The ***r***_*w*,*p*_ reported for additive genetic values for EGY was significantly higher for the DHGLM (0.9517) than for the LMM-HET (0.9467) in the bootstrap-based t-test (*P-value*< 0.01). The ***r***_*w*,*p*_ estimated for the additive genetic values in DGY was 0.9049; this value was significantly lower (~4%), in the t-test (*P-value*< 0.01) than predictions for additive genetic values for EGY from both models.

**Figure 6:**
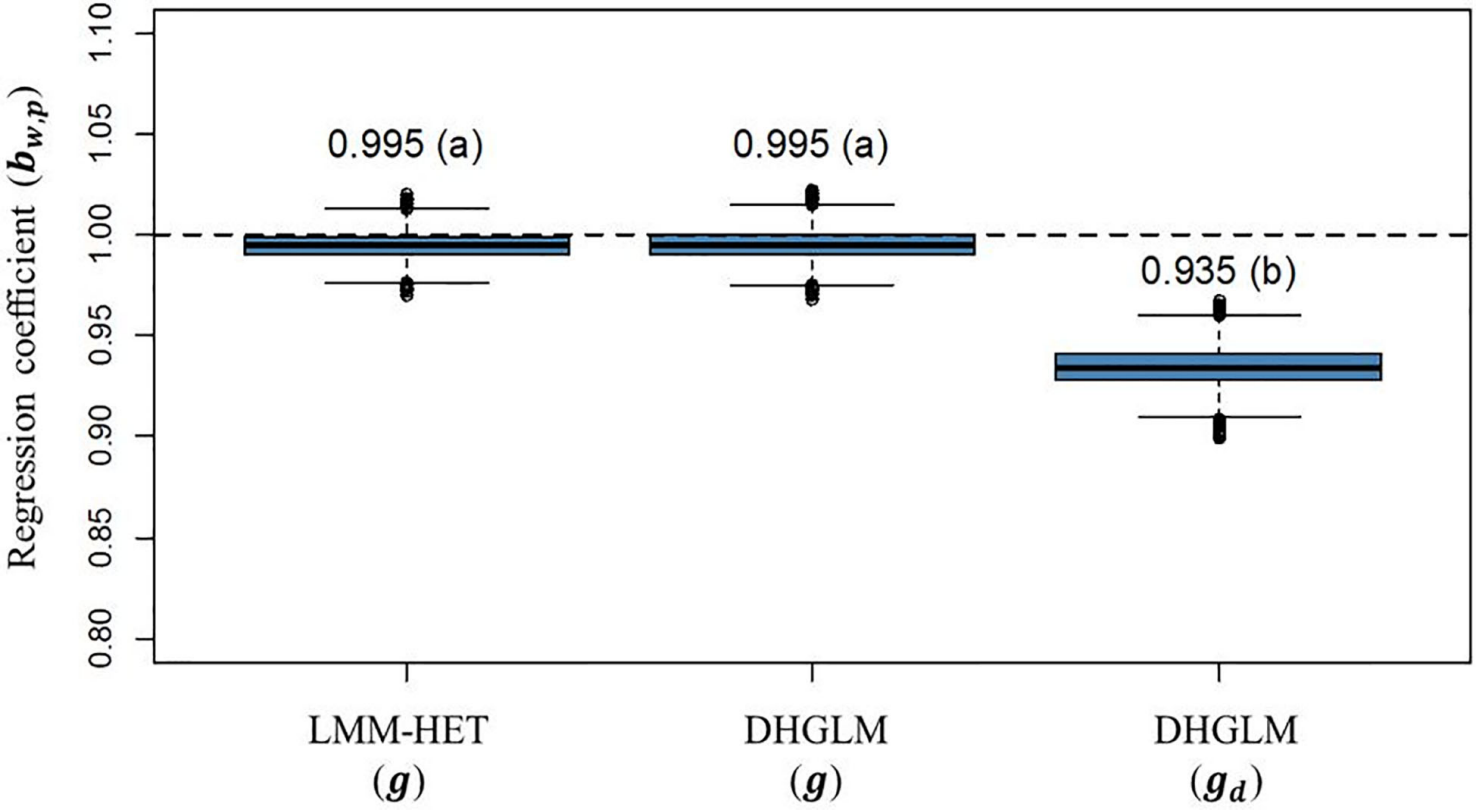
Boxplot of bootstrap distribution for the slope of the regression of additive genetic values obtained with whole phenotypic information on additive values obtained with partial phenotypic information (*b*_*w*,*p*_) in a leave-one-line-out (LOO) cross-validation. LMM-HET: linear mixed model allowing heteroscedasticity of residual variance for the different environments. DHGLM: double hierarchical generalized linear model. *g* : predicted additive genetic values for corrected phenotypes (*y*_*c*_). *g*_*d*_ : predicted additive genetic values for dispersion variable (*y*_*d*_). The letter above the bar denotes significant differences between models in a t-test (*P-value*< 0.01). The black dashed line represents a regression coefficient of one, where no under or over-dispersion is present.

The statistic for variance inflation of predicted additive genetic values (***b***_*w*,*p*_) are presented in **Figure 6**. The expected ***b***_*w*,*p*_ when there is no variance inflation in predictions is 1 (black dashed horizontal line in **Figure 6**); lower and higher values than the unity represent over or under-dispersion, respectively. In our study, we did not observe variance inflation in predictions for additive genetic values in EGY for the DHGLM (0.9951) and the LMM-HET (0.9955), and no significant difference between models was found. Conversely, the predictions for additive genetic values in DGY for the DHGLM revealed a low over-dispersion with a ***b***_*w*,*p*_ of 0.9346 and a bootstrap-based confidence interval ranging from 0.9159 to 0.9532. The ***b***_*w*,*p*_ for predictions of the additive genetic effects in EGY and DGY were significantly different in the t-test (*P-value*< 0.01).

## Discussion

In this study, we utilized a DHGLM to investigate the genetic variation in residual variance for grain yield (micro-environmental sensitivity) and to assess the predictability of additive genetic effects in EGY (***y*_*c*_**) and DGY (***y*_*d*_**) variables. As far as we know, studies on micro-environmental sensitivity have been limited in plants, and this is the first time that genetic parameters for micro-environmental sensitivity have been investigated for wheat grain yield. We found that the micro-environmental sensitivity for grain yield is heritable and that there is potential for reducing micro-environmental sensitivity and increasing resilience of wheat breeding lines. In addition, we found that the DHGLM had similar performance for predictions of additive genetic effects in EGY compared to the LMM-HET, and had suitable performance for prediction of additive genetic effects in DGY.

### Variance components and heritability estimates using LMM-HET and DHGLM

The LMM-HET and DHGLM revealed substantial variance for ***g***, ***l***, and ***f*** (**Table 2**). The ${\hat{\sigma }}_{\text{g}}^{2}$ had the same estimate for both models. Small differences between models were found for ${\hat{\sigma }}_{\text{l}}^{2}$ and ${\hat{\sigma }}_{\text{f}}^{2}$ , where a slightly higher ${\hat{\sigma }}_{\text{l}}^{2}$ and lower ${\hat{\sigma }}_{\text{f}}^{2}$ was observed for the DHGLM. The ${\hat{\sigma }}_{\text{l}}^{2}$ is the variance due to common line effects (***l***), which specify the genetic effects that are not captured by markers in the genomic term, and hence mostly non-additive variance is expected in ${\hat{\sigma }}_{\text{l}}^{2}$ . The residual variance ( ${\hat{\sigma }}_{{\text{e}}_{i}}^{2}$ ) was obtained for each year-location subset with both models, and generally similar values were obtained for the different environments. The largest differences were observed for environments #2 and #8 (**Table 2**), where the DHGLM had considerably lower ${\hat{\sigma }}_{{\text{e}}_{i}}^{2}$ . The lower residual variance is generally associated with a better fit of models, and in our case, it could be related to a more specific definition of heterogeneous residuals due to additive genetic effect in the DHGLM. The ${\hat{\sigma }}_{{\text{P}}_{i}}^{2}$ , $h_{i}^{2}$ and $H_{i}^{2}$ estimated with the LMM-HET and the DHGLM where in general similar across environments, except for environments #2 and #8 where the DHGLM had a considerable higher estimate of $h_{i}^{2}$ and $H_{i}^{2}$ . Despite the differences across environments, the $h_{i}^{2}$ and $H_{i}^{2}$ were in the range of previous studies for grain yield using Nordic Seed A/S data (Guo et al., 2020; Raffo et al., 2022a; Raffo et al., 2022b).

An alternative DHGLM was in addition implemented for VCs estimation to account for G*×*E due to additive-by-environment interactions in EGY. In this alternative formulation, the G*×*E effect was modelled using a genomic relationship matrix to relate lines within each environment. However, the G*×*E due to additive genotype-by-environment interactions was excluded from the analysis due to convergence problems in the REML algorithm. In this sense, the line × environment interaction effect (L×E) can be seen as a G*×*E effect capturing a combination of additive and non-additive effects, and helps to specify the G*×*E effects for our population.

The DHGLM was used to estimate fixed effects ( ${\hat{\text{b}}}_{d}$ ) and additive genetic variance in dispersion ( ${\hat{\sigma }}_{{\text{g}}_{d}}^{2}$ ). The ${\hat{\text{b}}}_{d}$ revealed a considerable variation, which implies that the specification of fixed effects in DGY is justified in the developed model. The fixed effects estimate the effect of trials nested within year-location subsets; therefore, they can capture variation in DGY due to differences in trials within the experimental field and across year-location environments. The genetic variance in DGY was ${\hat{\sigma }}_{{\text{g}}_{d}}^{2}$ = 0.004. Several authors have reported that using replications of genetically similar individuals (e.g. inbred lines, clones) can contribute to an accurate estimation of genetic effect in DGY (Mulder et al., 2007; Hill and Mulder, 2010; Iung et al., 2020). In this aspect, the analyzed population is a good source to obtain accurate estimates since several repetitions of each line were available, and there is a high genetic similarity between replications of F_6_ lines. The high genetic similarity among replications occurs due to the F_6_ lines originated from selfing of F_3:5_ lines, where segregation is very low as product of the high homozygosity due to previous generations of selfing (expected ~96.9%).

### Genetic parameters for micro-environmental sensitivity and implications for breeding activities

Understanding the genetics of micro-environmental sensitivity for grain yield in wheat can be useful to optimize breeding strategies and improve the adaptation of wheat cultivars to the environments where production is performed. The DHGLM allowed estimating genetic parameters associated with micro-environmental sensitivity such as $h_{d}^{2}$ and *GCV*_*E*_ which are discussed in this section.

The Mulder-Hill heritability of residual variance ( $h_{d}^{2}$ ) is defined as the regression coefficient of the additive genetic values for the dispersion variable on the squared phenotypic deviation (Mulder et al., 2007) as an analogy to the heritability in the classical sense (Falconer and Mackay, 1996). The $h_{d}^{2}$ can be interpreted as an indication of the proportion of the total variance that is explained by genetic variability in residual dispersion ( ${\hat{\sigma }}_{{\text{g}}_{d}}^{2}$ ). In general, $h_{d}^{2}$ estimates are expected to be low, and based on empirical studies, it has been in the range of<0.01 to 0.10 for different species (reviewed by Hill and Mulder, 2010, and Iung et al., 2020). In our study, we found a $h_{d}^{2}$ of 0.033, which is in the intermediate range reported in previous studies and implies that reducing micro-environmental sensitivity by selection is possible. For the interpretation of results, it is also relevant to consider that other potential sources of variation in DGY could affect the estimation of genetic parameters with an impact on decreasing or increasing the amount of residual genetic variance captured. For example, genetic variation due to G×E interactions could also be present at the level of DGY. To address this issue, we have initially included in the model a G×E interaction effect modeled with a genomic relationship matrix within each environment, and a L×E interaction effect (as used to model EGY), however, those effects were later excluded due to convergence problems in the REML algorithm. An additional source of variation on residuals that could affect genetic parameters is a scale effect for higher means inducing higher variances in residuals (Falconer and Mackay, 1996; Yang et al., 2011; Mulder et al., 2016). This issue is further discussed in the next section “*Genetic correlation between additive effects in EGY and DGY*”.

The genetic coefficient of variation (*GCV*_*E*_) allows inferring the potential for reducing micro-environmental sensitivity through selection (Mulder et al., 2007; Rönnegård et al., 2013; Mulder et al., 2016; Iung et al., 2017). Previous studies revealed large variation in the *GCV*_*E*_ across traits and species ranging from<0.01 to 0.86 (as reviewed by Sae-Lim et al., 2016 and Iung et al., 2020). In our population, we found a *GCV*_*E*_ of 0.061, which implies that reducing the average additive genetic values in dispersion in one unit of *GCV*_*E*_ will reduce the residual variance by 6.1% (Mulder et al., 2009). The *GCV*_*E*_ , is in addition, informative since (as the classical coefficient of variation) it can be compared across traits and species (Sonesson et al., 2013).

As revealed by $h_{d}^{2}$ and *GCV*_*E*_ , selecting lines with lower micro-environmental sensitivity is possible, and thus, it could be included within breeding goals to develop more resilient cultivars. This could be particularly valuable under future climate conditions where more variable and extreme weather events are expected. On the contrary, if genotype sensitivity is not considered for selection, and wheat lines are selected by their superior performance in specific environments, the selection could induce an indirect increase in sensitivity product of increasing the G×E component of selected lines (Falconer and Mackay, 1996).

### Genetic correlation between additive effects in EGY and DGY

The knowledge of the genetic correlation between additive effects in EGY and DGY (*ρ*_*g*,*g*_*d*__) is relevant to infer possible directions of micro-environmental sensitivity in breeding programs and guide selection decisions. A positive-sign *ρ*_*g*,*g*_*d*__ represent an unfavorable genetic correlation since it means that selecting for lines with higher additive value in EGY will result in higher micro-environmental sensitivity. In our study, we found an estimate of *ρ*_*g*,*g*_*d*__ of 0.502 (**Table 2**); however, several authors have argued that, to some extent, *ρ*_*g*,*g*_*d*__ can be influenced by scale effects, where higher variances in residuals are associated with higher means (Falconer and Mackay, 1996; Yang et al., 2011; Mulder et al., 2016). The scale effect may be present if the coefficient of variation of a trait remains constant as its mean change, then its variance must change as well (Walsh and Lynch, 2018). An alternative approach to evaluate *ρ*_*g*,*g*_*d*__ without the potential trend caused by scale effects is using the natural logarithm (ln) of the main response variable (YGD) to perform the analysis (Sonesson et al., 2013; Sae-Lim et al., 2015; Sae-Lim et al., 2017). The log-transformation reduces the dependency of variance on mean due to scale, and thus *ρ*_*g*,*g*_*d*__ is expected to be mainly influenced by genetic micro-environmental sensitivity and not by the scale effect.

To evaluate how the scale effect affects our population, we have performed an additional analysis using the natural logarithm (ln) of *y*_*c*_. The log-transformed *y*_*c*_ variable was observed normally distributed, and therefore the same model assumptions were made as for *y*_*c*_ . After log–transformation, we found a *ρ*_*g*,*g*_*d*__ value of -0.698. Similar results in terms of switching *ρ*_*g*,*g*_*d*__ from a positive to negative correlation after data transformation has been observed in previous studies for other species (Yang et al., 2011; Sae-Lim et al., 2015; Sae-Lim et al., 2017). The analysis based on ln (*y*_*c*_) is expected to yield a better estimate of *ρ*_*g*,*g*_*d*__ as it is free from distortions caused by the scale effect, and therefore, it is a more accurate measure of correlation due to genetic effects. The negative correlation found is favorable for reducing micro-environmental sensitivity in breeding programs since selection for higher additive values in EGY will result in lower additive values in DGY for log-transformed *y*_*c*_. In addition, we have observed a correlation close to 1 (0.99) between predictions for additive values in *y*_*c*_ and log-tranformed *y*_*c*_ . Based on these results, we concluded that select on both the EGY and DGY is possible and could represent a comprehensive strategy to increase grain yield and reduce micro-environmental sensitivity in winter wheat.

### Genomic prediction for grain yield and its micro-environmental sensitivity

The PA for predictions of additive genetic effect in EGY and DGY were evaluated in a leave-one-line-out (LOO) CV (**Figure 4**). No significant differences (*P-value* > 0.01) in PA for additive genetic effects in EGY were observed between the LMM-HET and DHGLM, and a similar trend was observed for the maximum potential PA (horizontal green lines in **Figure 4**). A small but significant improvement (*P-value*< 0.01) of 0.5% in ***r***_*w*,*p*_ was conferred by the DHGLM compared to the LMM-HET (**Figure 5**), and no variance inflation (***b***_*w*,*p*_) was observed for predictions of additive effect in EGY (predictions of ***g*** effect) for any of the proposed models (***b***_*w*,*p*_ values close to 1, **Figure 6**). The observed results revealed a good performance for predictions of additive genetic effect in EGY with both developed models. The good predictive performance for additive genetic effect in EGY in the DHGLM has also been observed in previous studies, especially when genomic information was used (Mulder et al., 2013a; Sell-Kubiak et al., 2015; Sae-Lim et al., 2017). In addition, the similar performance for prediction of breeding values for EGY using the LMM-HET and the DHGLM suggests that accounting for heterogeneous residuals by genotype in the DHGLM and the use of genetic correlations between additive effect in EGY and DGY in the DHGLM have not provided significant benefits for prediction.

The PA for additive genetic effects in DGY (predictions of *g*_*d*_ effect) was significantly lower than for additive genetic effect in EGY (**Figure 4**). This can be expected because given the low $h_{d}^{2}$ found, the potential PA of predicted additive values in dispersion is low (~0.48). The relationship between the trait’s heritability and prediction accuracy has been reported in the literature, where for lower values of heritability, lower accuracies are expected (Wimmer et al., 2013; Muranty et al., 2015). The ***r***_*w*,*p*_ estimate for prediction of additive effects in DGY was high (0.905); however, it was significantly lower (about 4%, *P-value*< 0.01) than for additive effects in EGY. The ***b***_*w*,*p*_ for predictions in DGY revealed a low over-dispersion for predicted values as observed in a ***b***_*w*,*p*_ value below one (0.935). A potential reason explaining over-dispersion could be related to the presence of other effects affecting DGY that could not be included in the model. For example, as earlier stated, genetic variation due to G×E interactions could be present at the level of DGY. However, those effects could not be included for our population due to problems of convergence with the REML algorithm.

## Limitations and prospects

The presented work can be seen as a proof of concept for studying micro-environmental sensitivity in crop breeding programs. Breeding program datasets could be reanalyzed in order to identify and select more resilient lines exhibiting lower micro-environmental sensitivity. Selecting for reduced micro-environmental sensitivity is a way to deal with/handle some specific elements of G×E. The variability at macro-environmental scale is another important source of G×E in crops. Future studies extending the proposed DHGLM to account for macro-environmental sensitivity could represent a comprehensive approach to reducing sensitivity at micro-and macro-environmental levels. Furthermore, this may provide a better understanding of the relationship between micro-and macro-environmental sensitivity, and hence more research in the area is justified.

In addition, other G×E related effects and spatial effects were initially attempted to be included in the DHGLM. However, we found limitations for algorithm convergence when increasing model complexity by adding these G×E effects and prohibitive computational times when modelling spatial effects. The prohibitive computational times could be due to the spatial effect and the additive genetic effect for DGY (***g*_*d*_**) partially competing for the same variation (i.e. confounding factors between the spatial effect at the observation plot and micro-environmental variation). Consequently, the spatial effects were accounted for in a two-step approach, first estimating them in a separated linear mixed model and second subtracting their estimates from the raw phenotypes. If confounding between spatial effect and micro-environmental sensitivity effectively occurs, either not correcting for the spatial effect could inflate the estimates for micro-environmental sensitivity, or correcting by them beforehand (i.e. as in our study) would lead to a conservative estimate of the variance of micro-environmental sensitivity. The conservative estimation in our study could imply a stronger micro-environmental sensitivity than we estimated, and therefore, it may be even more important for wheat breeding. Therefore, further work is useful in order to improve model inference.

## Conclusions

In this work, we used a double hierarchical generalized linear model (DHGLM) to study the micro-environmental sensitivity for grain yield in wheat. As far as we know, studies on micro-environmental sensitivity have been scarcely conducted in plants, and this is the first time that genetic parameters for micro-environmental sensitivity have been investigated in wheat. We found that the micro-environmental sensitivity for grain yield is heritable and that there is potential for its reduction, according to a Mulder-Hill heritability ( ${\hat{h}}_{d}^{2}$ ) of 0.033 and a genomic coefficient of variation (*GCV*_*E*_) of 0.061, respectively. The genetic correlation between additive effects for (expressed) grain yield and its residual dispersion (*ρ*_*g*,*g*_*d*__) was estimated, and we observed a correlation of 0.502. Further analysis using log-transformation of (expressed) phenotypes was performed to study a possible scale effect of higher dispersion variances induced by higher phenotypic values, and a negative genetic correlation was observed after transformation (-0.698). The estimate of *ρ*_*g*,*g*_*d*__ using the log-transformation is a more reliable estimate of genetic correlation as it is free from distortions caused by the scale effect. Based on these results, we concluded that breeding for reduced micro-environmental sensitivity is possible and can be included within breeding objectives without compromising the selection for increased yield. In addition, the double hierarchical generalized linear model had a good predictive performance for additive effects on residual dispersion, and showed similar performance for predicting additive genetic effects on (expressed) grain yield compared to a linear mixed model allowing for heteroscedasticity of residual variancep in different environments (LMM-HET). Such findings showed that the double hierarchical generalized linear models could be a good choice to predict additive genetic effects on (expressed) grain yield and its residual dispersion for wheat breeding.

## Data availability statement

The original contributions presented in the study are publicly available. This data can be found here: https://doi.org/10.7910/DVN/AB43I3.

## Author contributions

JJ and MR: conceptualization. MR and PS: data curation. MR: formal analysis. JJ, MR: funding acquisition. MR, JJ, BC, PS, RR, LM, TM-H: investigation, methodology, and project administration. JJ and PS: resources and supervision. MR, JJ: software, validation, visualization, and writing—original draft. JJ, BC, PS, RR, LM, TM-H: writing—review and editing. All authors contributed to the article and approved the submitted version.
