# Supplementary material for: Genomic prediction for grain yield and micro-environmental sensitivity in winter wheat
Source: Front Plant Sci. 2023 Feb 1;13:1075077. doi: 10.3389/fpls.2022.1075077 (PMC9929036; doi:10.3389/fpls.2022.1075077)
Supplement: Supplementary file 1 [file Table_1.docx]

**Supplementary material 1**

In this material we provide a formal statistical definition of the proposed Double Hierarchical Generalized Linear Model (DHGLM). Let $y_{ler}$ be the $r^{th}$ phenotypic measurement for the line $l$ in the environment $e$, then:

$$y_{ler}\sim N\left( \mu_{le}, \sigma_{le}^{2} \right), indpt$$

where $\mu_{le}$ and $\sigma_{le}^{2}$ are the mean and variance for the $r^{th}$ phenotypic measurement for line $l$ in the environment $e$, and $indpt$ denote independence between $\mu_{le}$ and $\sigma_{le}^{2}$. The mean ($\mu_{le}$) and variance ($\sigma_{le}^{2}$) are defined as:

$$\mu_{le}= x_{le}b+g_{l}+i_{l}+(ie)_{le}$$

$${log(\sigma}_{le}^{2})= x_{le}^{d}b_{l}^{d}+g_{l}^{d}$$

where $x_{le}b (x_{le}^{d}b_{l}^{d})$ represent fixed effect for the line $l$ in the environment $e$ for the mean (and dispersion); $g_{l}$ ($g_{l}^{d}$) represent the genetic additive effects for the line $l$ for the mean (and dispersion); $i_{l}$ represent the genetic effect due to uncorrelated line effect for the line $l$; $(ie)_{le}$ are the line × environment interaction effect (L×E) for the line $l$ in the environment $e$; and

$$\left( \begin{matrix} g \\ g^{d} \end{matrix} \right)\sim N\left( 0,\left( \begin{matrix} \sigma_{g}^{2} & \sigma_{g,g_{d}} \\ \sigma_{g,g_{d}} & \sigma_{g_{d}}^{2} \end{matrix} \right)\otimes\boldsymbol{G} \right)$$

$$i_{l}\sim N(0,\boldsymbol{I}\sigma_{l}^{2})$$

$${(ie)}_{le}\sim N(0,\boldsymbol{I}\sigma_{le}^{2})$$

where $\sigma_{g}^{2}$ and $\sigma_{g_{d}}^{2}$ are the genomic additive variance for mean and dispersion, respectively; $\sigma_{g,g_{d}}$ is the covariance between genetics effect in mean and dispersion; $\otimes$ denotes the Kronecker product; $\mathbf{G}$ is the GRM as defined for the LMM-HET model in the main manuscript; $\boldsymbol{I}$ is an identity matrix; $\sigma_{l}^{2}$ is the variance due to uncorrelated line effects; and $\sigma_{le}^{2}$ is the variance due to uncorrelated L×E effects.
