# Supplementary material for: Genomic prediction for grain yield and micro-environmental sensitivity in winter wheat
Source: Front Plant Sci. 2023 Feb 1;13:1075077. doi: 10.3389/fpls.2022.1075077 (PMC9929036; doi:10.3389/fpls.2022.1075077)
Supplement: Supplementary file 2 [file Table_2.docx]

**Supplementary material 2**

In this material, we present a plot showing the estimates for the fixed effects in dispersion $\boldsymbol{b}_{\boldsymbol{d}}$ (**Figure S1**).


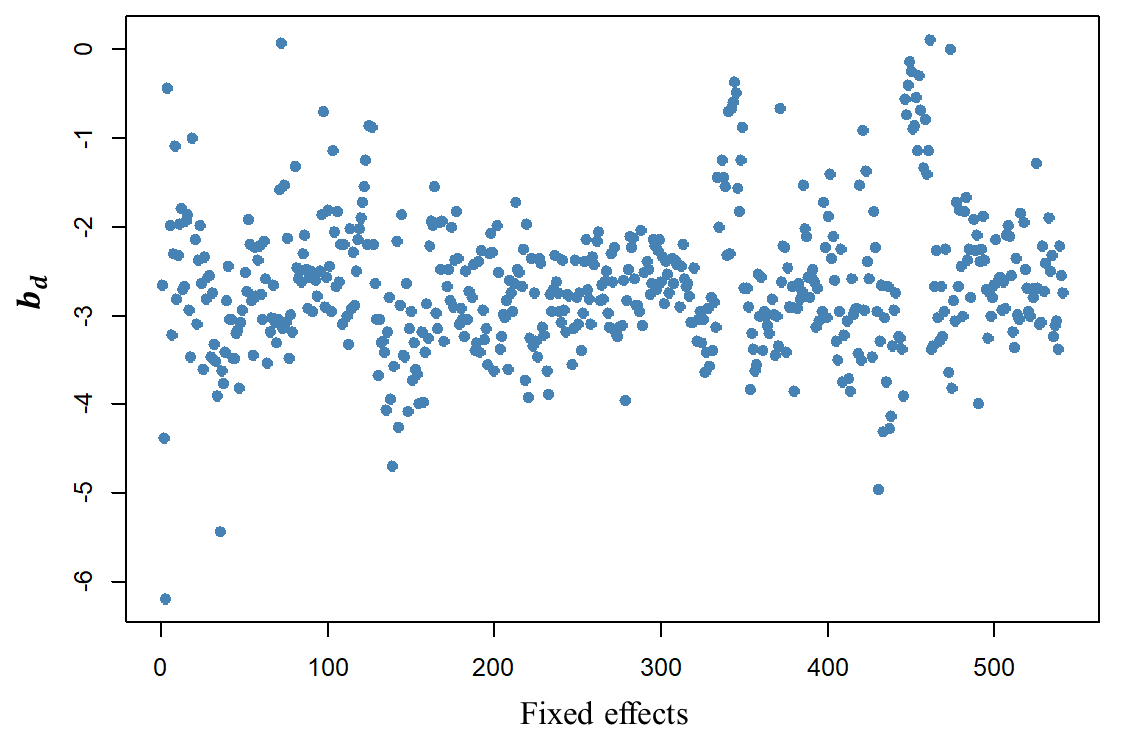
 **Figure S1.** Illustration of estimated fixed effect in dispersion $\boldsymbol{b}_{\boldsymbol{d}}$ for the double hierarchical generalized linear model (DHGLM).
